# Metabolic and Thyroid Alterations in HBV Monoinfection and HBV–HDV Coinfection: Insights from a Liver Transplant Cohort

**DOI:** 10.3390/metabo16100709

**Published:** 2026-09-24

**Authors:** Ayfer Serin, Safiye Koçulu Demir, Birkan Bozkurt, Ender Anılır, İsmail Sivri, Yaman Tokat

**Affiliations:** 1 Department of Gastroenterology, University of Health Sciences, Bağcılar Training and Research Hospital, Istanbul 34200, Türkiye; 2 Department of Infectious Diseases and Clinical Microbiology, Sancaktepe Şehit Prof. Dr. İlhan Varank Training and Research Hospital, Sancaktepe 34000, Türkiye; 3 Department of General Surgery, Başakşehir Çam and Sakura City Hospital, Başakşehir 34480, Türkiye; 4 Department of General Surgery, Ödemiş State Hospital, Ödemiş 35750, Türkiye; 5 Department of Anatomy, Faculty of Medicine, Kocaeli University, Umuttepe Campus, İzmit 41001, Türkiye; 6 General Surgery, Acıbadem Health Group, Istanbul 34394, Türkiye

**Keywords:** hepatitis B virus, hepatitis D virus, HDV RNA, triglycerides, thyroid function, liver transplantation

## Abstract

Background: Metabolic alterations associated with hepatitis B virus (HBV)–hepatitis D virus (HDV) coinfection remain poorly characterized. We investigated metabolic and endocrine profiles in HBV monoinfection and HBV–HDV coinfection, with emphasis on quantitative HDV activity. Methods: This retrospective observational study included HBsAg-positive adult liver transplant candidates. Pre-transplant metabolic, biochemical, thyroid, and virological parameters were evaluated. Associations between quantitative HDV RNA and metabolic parameters were assessed using Spearman correlation and multivariable regression. Results: Conventional circulating metabolic parameters were broadly comparable between groups. HBV–HDV coinfection was associated with higher adjusted odds of diabetes mellitus, although this association was attenuated after accounting for liver disease severity. TSH was independently lower in HBV–HDV coinfection, whereas FT3 and FT4 did not differ independently. Among patients with HBV–HDV coinfection, HDV RNA correlated positively with triglycerides (ρ = 0.437, *p* = 0.009). Each 1-log10 increase in HDV RNA was associated with a 5.42 mg/dL increase in triglycerides after adjustment for HBV DNA, age, sex, and MELD score (95% CI 1.84–9.00; *p* = 0.003), with consistent associations in BMI- and diabetes-adjusted sensitivity models. Conclusions: Higher quantitative HDV RNA was independently associated with higher triglyceride concentrations, identifying a potential viral–metabolic interaction that warrants prospective validation.

## 1. Introduction

Chronic hepatitis B virus (HBV) infection remains a major global cause of chronic liver disease, cirrhosis, hepatic decompensation, hepatocellular carcinoma (HCC), and liver-related mortality [1]. Hepatitis D virus (HDV) is a defective RNA virus that requires hepatitis B surface antigen for virion assembly and propagation, and chronic HBV–HDV coinfection represents one of the most aggressive forms of viral hepatitis [2]. Compared with HBV monoinfection, chronic hepatitis D is associated with accelerated fibrosis progression, earlier development of cirrhosis, increased hepatic decompensation, and a substantial burden of liver-related complications [2,3,4]. Recent longitudinal cohorts further demonstrate that detectable HDV RNA is associated with an increased risk of severe liver-related outcomes, supporting the clinical relevance of ongoing HDV replication beyond the diagnosis of coinfection itself [3,4].

The biological consequences of chronic viral hepatitis extend beyond direct hepatocellular injury. The liver is a central regulator of systemic metabolic homeostasis, integrating glucose utilization and storage, lipid synthesis and oxidation, cholesterol metabolism, bile acid turnover, amino acid metabolism, mitochondrial bioenergetics, and endocrine signaling. Chronic viral infection may interfere with these pathways through direct viral effects on hepatocytes as well as through persistent inflammation, oxidative stress, immune activation, and mitochondrial dysfunction. Experimental and translational evidence indicates that HBV can reprogram multiple host metabolic pathways, including glycolysis, the tricarboxylic acid cycle, oxidative phosphorylation, gluconeogenesis, fatty-acid metabolism, and cholesterol homeostasis [5]. Such metabolic reprogramming may facilitate viral persistence while simultaneously altering immune-cell function and the hepatic microenvironment [5].

Glucose and lipid metabolism are particularly relevant in chronic HBV infection. Alterations in glucose handling, insulin signaling, hepatic lipid processing, and mitochondrial energy metabolism have increasingly been recognized as components of the complex interaction between HBV and its host [5,6,7,8]. Metabolic comorbidities may coexist with chronic HBV infection and potentially influence fibrosis progression and HCC risk [6,7,8]. Nevertheless, most available evidence regarding viral–metabolic interactions has focused on HBV monoinfection or on the coexistence of HBV with metabolic dysfunction-associated steatotic liver disease. Considerably less is known about the metabolic phenotype associated specifically with HBV–HDV coinfection.

Circulating biochemical parameters may provide clinically accessible indicators of these interactions. Fasting glucose, insulin, and the homeostatic model assessment of insulin resistance (HOMA-IR) reflect different components of glucose–insulin homeostasis, whereas triglycerides and cholesterol are closely linked to hepatic lipid synthesis, transport, and clearance. Bilirubin and creatinine are not specific markers of metabolic dysfunction; nevertheless, they are circulating biochemical products closely related to hepatic processing, systemic homeostasis, and the severity of advanced liver disease. Simultaneous evaluation of these parameters may therefore provide a broader description of the metabolic environment accompanying chronic HBV and HBV–HDV infection.

Importantly, metabolic differences between HBV monoinfection and HBV–HDV coinfection may not necessarily be captured by simple group comparisons. The presence of HDV identifies coinfection, whereas quantitative HDV RNA provides information regarding active viral replication. In a recent multicenter cohort, HDV RNA positivity was independently associated with severe liver-related outcomes [3]. Similarly, a large 2026 longitudinal analysis of 565 patients with chronic hepatitis D showed that detectable HDV RNA was associated with liver-related events, with risk increasing at HDV RNA levels of at least 1000 IU/mL [4]. Whether the magnitude of HDV viremia is also associated with circulating metabolic parameters remains much less clearly established. Evaluating quantitative HDV RNA alongside HBV DNA may therefore provide additional insight into whether metabolic alterations are related to viral activity rather than merely to the presence of coinfection.

This question is particularly relevant for lipid and glucose metabolism because the liver is simultaneously the major site of HBV and HDV-related disease and a principal organ governing systemic lipid and carbohydrate homeostasis. A relationship between quantitative viral activity and circulating triglyceride, cholesterol, or glucose concentrations could therefore provide a clinically measurable signal of host–virus metabolic interaction. Such associations would not establish a direct mechanistic effect of viral replication, particularly in advanced liver disease where disease severity, nutritional status, body composition, and endocrine factors may also influence circulating metabolites. However, they could identify metabolic patterns suitable for validation in prospective and mechanistic studies.

The thyroid–liver axis represents another important component of systemic metabolic homeostasis. Thyroid hormones regulate hepatic lipid synthesis and oxidation, cholesterol metabolism, carbohydrate metabolism, mitochondrial activity, and energy expenditure, whereas the liver contributes to thyroid hormone transport, activation, inactivation, and clearance [9,10,11,12,13]. Contemporary mechanistic reviews emphasize that thyroid hormone receptor-β signaling is particularly important in hepatic lipid homeostasis and that disruption of thyroid signaling can contribute to hepatic lipid accumulation, insulin resistance, inflammation, and fibrosis [9,10,11,12,13]. The recent development of liver-directed thyroid hormone receptor-β agonism for metabolic liver disease further illustrates the biological importance of this pathway [9,12,13].

These interactions are especially relevant when interpreting thyroid-stimulating hormone (TSH), free triiodothyronine (FT3), and free thyroxine (FT4) in patients with advanced liver disease. Changes in circulating thyroid parameters may reflect altered hepatic hormone metabolism, systemic illness, nutritional status, inflammatory activity, or changes in the hypothalamic–pituitary–thyroid axis. At the same time, thyroid hormone signaling itself influences lipid and glucose homeostasis [9,10,11,12,13]. Thus, assessment of thyroid parameters together with conventional metabolic markers may provide a more integrated characterization of the metabolic–endocrine phenotype associated with HBV–HDV coinfection.

HCC adds another layer of complexity to the interpretation of biochemical and metabolic profiles in chronic viral liver disease. HBV and HDV are both associated with HCC risk [1,2], while alpha-fetoprotein (AFP) remains a clinically relevant complementary biomarker when interpreted together with imaging and other clinical information [14,15]. AFP concentrations, however, may also increase in the absence of HCC as a consequence of hepatocellular injury, regeneration, and inflammatory activity. Therefore, assessment of AFP according to HCC status may help distinguish tumor-related changes from biochemical differences associated with the underlying viral disease.

Despite growing knowledge regarding HBV-induced metabolic reprogramming and the clinical importance of HDV viremia, integrated studies evaluating glucose–insulin homeostasis, circulating lipid-related parameters, thyroid function, and quantitative viral markers in HBV–HDV coinfection remain limited. In particular, the relationship between quantitative HDV RNA and conventional circulating metabolic parameters has not been well characterized. This represents a clinically relevant knowledge gap because identification of viral-load-associated metabolic patterns could provide new insights into host–virus interactions and generate hypotheses for subsequent mechanistic and prospective studies.

Accordingly, the present study aimed to characterize metabolic and endocrine profiles in patients with HBV monoinfection and HBV–HDV coinfection in a liver transplant cohort. We compared glucose–insulin homeostasis, conventional circulating metabolic parameters, and thyroid function between the two groups and assessed whether observed associations persisted after adjustment for relevant clinical factors. We further investigated the relationships of quantitative HDV RNA and HBV DNA with metabolic parameters, with particular attention to lipid and glucose metabolism. Finally, AFP patterns were evaluated according to HCC status to provide additional context for the biochemical differences observed between HBV monoinfection and HBV–HDV coinfection.

## 2. Materials and Methods

### 2.1. Study Design and Population

This retrospective observational cohort study included adult patients with HBV-related chronic liver disease who underwent liver transplantation at the Şişli Florence Nightingale Hospital/Demiroğlu Bilim University liver transplantation center in Istanbul, Türkiye, between January 2004 and December 2019. Patients were classified into HBV monoinfection and HBV–HDV coinfection groups according to documented HBV/HDV infection status. The study population comprised patients with available pre-transplant clinical, biochemical, metabolic, endocrine, and virological data. Pediatric patients and patients without sufficient.

Information used to determine HBV/HDV infection status was excluded from comparative analyses. Complete-case analysis was used for individual laboratory, correlation, and regression analyses; therefore, the effective sample size varied according to the availability of variables required for each analysis. All variables evaluated in the present study were obtained during the pre-transplant assessment period and represented the clinical and biochemical status before liver transplantation.

### 2.2. Definition of HBV and HBV–HDV Infection

All patients included in the study were HBsAg-positive and had HBV-related chronic liver disease. Patients were classified as having HBV monoinfection or HBV–HDV coinfection according to documented HDV status. HBV–HDV coinfection was identified on the basis of documented anti-HDV positivity together with a clinical history of chronic delta infection. Patients without evidence of HDV infection were classified as having HBV monoinfection. Available quantitative HBV DNA measurements were used to characterize HBV replication. Quantitative HDV RNA measurements, when available, were analyzed separately as a continuous virological variable to characterize active HDV replication [3]. This approach allowed HDV infection status and quantitative viral activity to be evaluated separately.

### 2.3. Clinical and Metabolic Variables

Demographic and clinical variables included age, sex, body mass index (BMI), diabetes mellitus, and indices of liver disease severity. BMI was calculated as body weight in kilograms divided by height in meters squared (kg/m^2^). Pre-transplant biochemical and metabolic parameters included fasting glucose, glycated hemoglobin (HbA1c), fasting insulin, total cholesterol, triglycerides, total bilirubin, creatinine, albumin, aminotransferases, gamma-glutamyl transferase (GGT), alkaline phosphatase (ALP), international normalized ratio (INR), sodium, and platelet count, according to availability in the clinical records. Insulin resistance was estimated using the homeostatic model assessment of insulin resistance (HOMA-IR), calculated as fasting glucose (mg/dL) × fasting insulin (µIU/mL)/405 [16]. Only paired fasting glucose and insulin measurements suitable for calculation were included in HOMA-IR analyses. Liver disease severity was evaluated using the Model for End-Stage Liver Disease (MELD) score [17]. Child–Pugh classification was additionally considered in sensitivity analyses where specified.

### 2.4. Timing of Pre-Transplant Laboratory and Virological Assessments

For living donor liver transplantation, routine pre-transplant laboratory tests were repeated one day before transplantation, and the most recent values were used for analysis. For deceased donor liver transplantation, routine laboratory tests were obtained one day before transplantation. Thyroid function tests (TSH, FT3, and FT4) and lipid parameters, including triglycerides and total cholesterol, were assessed within 1 day to 1 month before transplantation; when multiple measurements were available, the value closest to the transplantation date was selected. HBV DNA and HDV RNA measurements were updated within a maximum of 3 months before transplantation, and the most recent available pre-transplant measurement was used.

### 2.5. Thyroid Function Assessment

Pre-transplant thyroid parameters included thyroid-stimulating hormone (TSH), free triiodothyronine (FT3), and free thyroxine (FT4). Because laboratory reporting conventions changed during the long study period, FT4 values were harmonized to a common unit before statistical analysis. TSH, FT3, and FT4 were evaluated as continuous variables in between-group comparisons and multivariable analyses examining their relationships with HBV/HDV infection status and relevant clinical and metabolic factors.

### 2.6. Virological and Metabolic Analyses

Among patients with HBV–HDV coinfection, quantitative HDV RNA was examined in relation to fasting glucose, total cholesterol, triglycerides, total bilirubin, and creatinine. Because viral-load measurements showed markedly skewed distributions, quantitative HDV RNA and HBV DNA were transformed as log10(value + 1) for regression analyses. The association between HDV RNA and triglyceride concentration was further examined using multivariable linear regression. The principal model included HDV RNA, HBV DNA, age, sex, and MELD score. Additional sensitivity models separately incorporated BMI and diabetes mellitus to assess the robustness of the association to these metabolic factors. Analyses were restricted to patients with quantitative HDV RNA measurements and complete data for the variables included in each model; therefore, sample sizes differed between correlation and multivariable analyses. As additional robustness analyses, triglyceride concentration was natural-log-transformed and the principal model was repeated with log-transformed triglycerides as the outcome. The principal HDV RNA–triglyceride model was also evaluated using 5000 bootstrap resamples with percentile-based 95% confidence intervals. Because quantitative HDV RNA was not available for all coinfected patients, availability of HDV RNA was examined descriptively, including comparisons between patients with and without an available HDV RNA result. An exploratory analysis restricted to patients with detectable/positive HDV RNA was also performed; because of the limited subgroup size, no high-dimensional adjusted model was fitted in this subgroup.

### 2.7. AFP and HCC Assessment

The presence of hepatocellular carcinoma (HCC) before transplantation was determined from the pre-transplant clinical record on the basis of an established radiological and/or pathological diagnosis documented during transplant evaluation [14]. Serum alpha-fetoprotein (AFP) concentrations were analyzed according to HBV/HDV infection status and stratified by the presence or absence of HCC. Because AFP may be elevated in chronic liver disease independently of HCC, additional analyses were performed among patients without HCC. Within the non-HCC subgroup, correlations between AFP and liver-related biochemical parameters, including GGT, AST, ALT, ALP, albumin, INR, total bilirubin, platelet count, and MELD score, were assessed. Multivariable models were subsequently used to evaluate whether differences in AFP between HBV monoinfection and HBV–HDV coinfection persisted after adjustment for relevant clinical and biochemical factors.

### 2.8. Statistical Analysis

Continuous variables were assessed for distributional characteristics and are presented primarily as median and interquartile range (IQR). Categorical variables are reported as numbers and percentages. Between-group comparisons of continuous variables were performed using the Mann–Whitney U test, whereas categorical variables were compared using appropriate contingency-table methods. Associations between quantitative HDV RNA and metabolic parameters, as well as correlations involving AFP, were evaluated using Spearman rank correlation coefficients. Multivariable logistic regression was used for binary outcomes, including diabetes mellitus. Continuous outcomes with skewed distributions were natural-log-transformed when appropriate, and effect estimates from these models are presented as geometric mean ratios with 95% confidence intervals (CIs). For triglyceride concentrations according to HDV RNA level, multivariable linear regression models used log10-transformed HDV RNA and HBV DNA. The principal model was adjusted for age, sex, HBV DNA, and MELD score; BMI and diabetes mellitus were incorporated separately in sensitivity analyses to limit model complexity in the available complete-case sample. Heteroskedasticity-consistent HC3-robust standard errors were used in multivariable linear models. For the principal HDV RNA–triglyceride association, robustness was additionally assessed using a natural-log-transformed triglyceride outcome and 5000 bootstrap resamples with percentile-based 95% confidence intervals. Sensitivity analyses of the association between HBV–HDV status and TSH additionally considered platelet count, albumin, sodium, and MELD score. Missing observations were not imputed; each analysis was based on patients with complete data for the variables included in the corresponding model, and effective sample sizes are reported in the tables. All statistical tests were two-sided, and *p* < 0.05 was considered statistically significant. Given the exploratory nature of several secondary and viral-load analyses, effect estimates, confidence intervals, consistency across sensitivity analyses, and biological plausibility were considered together when interpreting the findings. All statistical analyses were performed using the Statistical Package for the Social Sciences (SPSS), version 14.0 (SPSS Inc., Chicago, IL, USA).

### 2.9. Ethical Considerations

The study was conducted in accordance with the principles of the Declaration of Helsinki and was approved by the Ethics Committee of Demiroğlu Bilim University (21 July 2020; Decision No. 2020-13-02).

## 3. Results

### 3.1. Clinical, Metabolic, and Endocrine Characteristics

Patients with HBV–HDV coinfection were younger than those with HBV monoinfection (median 48.0 vs. 53.0 years, *p* < 0.001). BMI and fasting glucose levels were comparable between the groups. Diabetes mellitus was numerically more frequent in the HBV–HDV group (24.5% vs. 16.8%), although the unadjusted difference was not statistically significant (*p* = 0.120). Similarly, insulin concentrations and HOMA-IR tended to be higher in patients with HBV–HDV coinfection, but the differences did not reach statistical significance (insulin, *p* = 0.070; HOMA-IR, *p* = 0.155) (Table 1).

Conventional circulating metabolic parameters, including total bilirubin, creatinine, total cholesterol, and triglycerides, did not differ significantly between HBV monoinfection and HBV–HDV coinfection. Median triglyceride concentrations were 70.0 mg/dL (IQR 57.0–97.0) in the HBV group and 76.0 mg/dL (IQR 63.0–98.0) in the HBV–HDV group (*p* = 0.170). Likewise, no significant between-group differences were observed for total bilirubin (*p* = 0.117), creatinine (*p* = 0.168), or total cholesterol (*p* = 0.502) (Table 1).

Among thyroid parameters, TSH was significantly lower in the HBV–HDV group than in the HBV monoinfection group (median 1.44 vs. 1.71, *p* = 0.011). FT3 showed a nonsignificant tendency toward higher values in the HBV–HDV group (*p* = 0.088), whereas standardized FT4 concentrations were comparable between the groups (*p* = 0.672). The median MELD score was 16.0 in HBV–HDV coinfection and 14.0 in HBV monoinfection (*p* = 0.050) (Table 1).

### 3.2. Adjusted Metabolic and Endocrine Associations with HBV–HDV Coinfection

After adjustment for age, sex, MELD score, and BMI, HBV–HDV coinfection was associated with higher odds of diabetes mellitus (adjusted OR 2.075, 95% CI 1.057–4.076; *p* = 0.034). However, this association was attenuated in the Child–Pugh sensitivity analysis (adjusted OR 1.890, 95% CI 0.919–3.885; *p* = 0.084).

The adjusted associations with HOMA-IR and insulin were of borderline statistical significance. HBV–HDV coinfection was associated with a HOMA-IR ratio of 1.243 (95% CI 0.989–1.562; *p* = 0.062) and an insulin ratio of 1.205 (95% CI 0.988–1.470; *p* = 0.065) (Table 2).

In contrast, the association with TSH remained statistically significant after multivariable adjustment. HBV–HDV coinfection was associated with a lower adjusted TSH level (ratio 0.712, 95% CI 0.579–0.876; *p* = 0.001). No independent associations were observed for FT3 (ratio 1.077, 95% CI 0.953–1.218; *p* = 0.236) or FT4 (ratio 1.006, 95% CI 0.928–1.090; *p* = 0.884) (Table 2).

The association between HBV–HDV coinfection and lower TSH remained consistent across alternative adjustment models incorporating platelet count, albumin, sodium, and MELD score, with HBV–HDV/HBV ratios ranging from 0.691 to 0.726 (all *p* ≤ 0.002) (Supplementary Table S1).

### 3.3. HDV RNA Levels and Metabolic Parameters

We next explored whether metabolic parameters were associated with the magnitude of HDV viremia among patients with HBV–HDV coinfection. HDV RNA levels showed a positive correlation with triglyceride concentrations (Spearman ρ = 0.437, *p* = 0.009; *n* = 35) (Figure 1). A weaker positive correlation was observed between HDV RNA and fasting glucose (ρ = 0.328, *p* = 0.034; *n* = 42). In contrast, HDV RNA was not significantly correlated with total bilirubin, creatinine, or total cholesterol (Table 3).

The association between HDV RNA and triglyceride concentrations persisted after multivariable adjustment. In a model including HBV DNA, age, sex, and MELD score, each 1-log10 increase in HDV RNA was associated with a 5.42 mg/dL higher triglyceride concentration (95% CI 1.84–9.00; *p* = 0.003; *n* = 32).

The association remained significant when BMI was additionally included in the model (B = 5.33, 95% CI 1.53–9.14; *p* = 0.006; *n* = 29) and in a separate sensitivity model additionally accounting for diabetes mellitus (B = 5.32, 95% CI 0.23–10.41; *p* = 0.041; *n* = 25) (Table 3).

The association was also robust to alternative outcome specification. When triglyceride concentration was natural-log-transformed, higher HDV RNA remained significantly associated with higher triglyceride levels (*p* = 0.0051), corresponding to an estimated 6.5% increase in triglycerides per 1-log10 increase in HDV RNA (95% CI approximately 1.9–11.3%). In the 5000-resample bootstrap analysis of the principal model, the percentile 95% confidence interval for the HDV RNA coefficient was 1.74–9.26 mg/dL, and 99.6% of bootstrap coefficients were positive. These sensitivity analyses are summarized in Supplementary Figure S1.

Quantitative HDV RNA results were available in 42 of 104 patients with HBV–HDV coinfection and unavailable in 62. Of the 42 patients with an available result, 23 had detectable/positive HDV RNA and 19 had a zero/negative result. Patients with and without an available HDV RNA result did not show statistically significant differences in age, MELD score, triglycerides, BMI, TSH, FT3, HbA1c, or diabetes mellitus (Supplementary Table S2). HCC, however, was less frequent among patients with an available HDV RNA result (1/42 [2.4%] vs. 11/62 [17.7%], *p* = 0.025), indicating potential selection related to HDV RNA availability. In an exploratory model additionally including HCC, the HDV RNA–triglyceride association remained similar (B = 5.49 mg/dL per 1-log10 increase, 95% CI 1.82–9.16; *p* = 0.0034); this model should be interpreted cautiously because only one patient with HCC was represented among those with available HDV RNA. The flow of quantitative HDV RNA availability and complete-case analyses is shown in Figure 2.

Flow diagram showing the HBV monoinfection and HBV–HDV coinfection groups, availability of quantitative HDV RNA measurements within the HBV–HDV group, and effective complete-case sample sizes for the HDV RNA–triglyceride analyses. Quantitative HDV RNA was available in 42 of 104 patients with HBV–HDV coinfection and unavailable in 62; 23 of the 42 available results were detectable/positive and 19 were recorded as zero/negative. Missing values were not imputed. The Spearman analysis included 35 paired HDV RNA–triglyceride observations; the principal, BMI-adjusted, and diabetes-adjusted regression models included 32, 29, and 25 patients, respectively.

When the analysis was restricted to patients with detectable/positive HDV RNA and available triglycerides (*n* = 20), the correlation remained positive in direction (Spearman ρ = 0.351) but was not statistically significant (*p* = 0.130). The small subgroup size was considered insufficient for a reliable multivariable model with the full covariate set.

Thus, although overall triglyceride concentrations did not differ significantly between the HBV and HBV–HDV groups, higher HDV RNA levels within the coinfected group were associated with higher circulating triglyceride concentrations.

### 3.4. AFP According to HCC Status

AFP concentrations differed according to HBV/HDV status and the presence of HCC. In the overall cohort, median AFP was higher in patients with HBV–HDV coinfection than in those with HBV monoinfection (9.80 vs. 6.02, *p* = 0.029). Among patients without HCC, this difference was more pronounced (12.59 vs. 5.29, *p* < 0.001). In contrast, among patients with HCC, AFP was numerically lower in the HBV–HDV group, although the difference did not reach statistical significance (5.31 vs. 11.25, *p* = 0.060) (Table 4).

Among patients without HCC, AFP showed positive correlations with GGT (ρ = 0.245, *p* < 0.001) and AST (ρ = 0.226, *p* < 0.001), whereas no significant correlations were observed with total bilirubin, platelet count, or MELD score. In multivariable analyses restricted to patients without HCC, AFP remained higher in HBV–HDV coinfection across the prespecified adjustment models, with adjusted HBV–HDV/HBV AFP ratios ranging from 2.788 to 3.030 (all *p* < 0.001) (Table 4).

## 4. Discussion

The present study provides an integrated assessment of metabolic, endocrine, and virological characteristics in HBsAg-positive liver transplant candidates with HBV monoinfection or HBV–HDV coinfection. The principal finding was that quantitative HDV RNA was positively associated with circulating triglyceride concentrations within the coinfected group, despite the absence of a significant difference in median triglyceride levels between the two infection groups. This association persisted after adjustment for HBV DNA, age, sex, and MELD score and remained significant in sensitivity models additionally accounting for BMI or diabetes mellitus. HBV–HDV coinfection was also associated with lower TSH concentrations, whereas FT3 and FT4 did not differ independently. The adjusted association with diabetes was attenuated after accounting for liver disease severity, and AFP differences persisted among patients without HCC. Together, these findings suggest that viral activity may reveal metabolic signals that are not apparent from comparisons based solely on infection status.

### 4.1. HDV Replication and Lipid Metabolism

The association between quantitative HDV RNA and triglyceride concentration is the most distinctive metabolic finding of this study. Increasing HDV RNA correlated with higher triglyceride concentrations within the HBV–HDV group (Spearman ρ = 0.437), and the association remained independently significant after adjustment for HBV DNA, age, sex, and MELD score. Its persistence after additional adjustment for BMI or diabetes mellitus reduces the likelihood that these common metabolic factors alone explain the observed relationship.

The consistency of this signal across the log-triglyceride sensitivity analysis and bootstrap resampling provides additional support for its robustness within the available sample. Nevertheless, the analysis restricted to patients with detectable HDV RNA was underpowered and did not reach statistical significance, despite retaining a positive direction of association. These findings therefore support a graded association in the analyzed sample but should not be interpreted as definitive evidence of a dose–response relationship among viremic patients alone.

This distinction between coinfection status and quantitative viral activity may be biologically relevant. Contemporary cohorts increasingly demonstrate that detectable or higher HDV RNA identifies clinically meaningful differences among patients with chronic hepatitis D. d’Almeida et al. reported a higher risk of severe liver-related outcomes in HDV RNA-positive patients [3], while the D-SOLVE and HDV-1000 analysis similarly showed that detectable HDV RNA was independently associated with liver-related events [4]. These studies did not evaluate triglyceride metabolism specifically, but they support the broader concept that quantitative HDV activity may capture biological information beyond HDV serostatus.

Direct clinical evidence linking HDV RNA concentration to circulating triglyceride levels remains limited. Accordingly, the present association should not be interpreted as evidence that HDV replication directly causes hypertriglyceridemia. The broader HBV literature nevertheless provides biological plausibility for a viral–metabolic interaction. HBV can reprogram host glucose and lipid pathways, including glycolysis, mitochondrial energy metabolism, fatty-acid metabolism, and lipid homeostasis [5,6,7,8]. Whether HDV modifies these HBV-associated pathways is not well established. The absence of a significant overall triglyceride difference between HBV monoinfection and HBV–HDV coinfection is therefore not inconsistent with the within-HDV viral-load association; the latter may represent a viral-activity-related metabolic gradient rather than a generalized lipid phenotype of coinfection.

HDV RNA should not, however, be considered a universal surrogate for all biological consequences of chronic hepatitis D. A recent European multicenter cohort found that HDV RNA levels were less informative for progression to cirrhosis than non-invasive measures such as liver stiffness and FIB-4 [18]. Thus, the HDV RNA–triglyceride relationship observed here is best regarded as a specific, hypothesis-generating metabolic signal requiring independent validation.

### 4.2. Glucose Metabolism, Insulin Resistance, and Diabetes

HBV–HDV coinfection was associated with approximately two-fold higher adjusted odds of diabetes mellitus, while insulin and HOMA-IR showed borderline associations. However, the diabetes association was attenuated and no longer statistically significant when Child–Pugh status was considered. This finding is important because advanced liver dysfunction itself can substantially alter glucose and insulin homeostasis. Current literature also indicates that interactions between chronic HBV infection and metabolic dysfunction are heterogeneous rather than uniformly additive [5,6,7,8].

The positive correlation between HDV RNA and fasting glucose provides an additional metabolic signal, but it was weaker than the triglyceride association and arose from an exploratory analysis. Taken together, the present data do not establish a distinct HDV-associated insulin-resistant phenotype. They instead suggest that glucose–insulin abnormalities in advanced HBV–HDV disease may reflect a combination of viral, hepatic, and host metabolic factors, with stronger evidence in this cohort for the HDV RNA–triglyceride relationship than for an independent effect on insulin resistance.

### 4.3. Thyroid–Liver Axis in HBV–HDV Coinfection

Lower TSH concentrations represented a second consistent finding. The association persisted after multivariable adjustment and across sensitivity models incorporating markers of liver disease severity, whereas FT3 and FT4 did not differ independently. The thyroid–liver axis is closely integrated with systemic metabolism: thyroid hormones regulate hepatic lipid oxidation, lipogenesis, cholesterol turnover, glucose metabolism, mitochondrial activity, and energy expenditure, while the liver contributes to thyroid hormone transport and metabolism [9,10,11,12,13].

Thyroid abnormalities in advanced liver disease are heterogeneous. Classical studies have described altered thyroid tests despite clinical euthyroidism [19], and a previous study in chronic hepatitis D reported associations between thyroid autoantibodies, transaminase activity, and HDV RNA, although overt thyroid dysfunction was uncommon [20]. Our population and endpoints differ from that study. Therefore, lower TSH without independent FT3 or FT4 differences should not be interpreted as primary thyroid disease or as a generalized thyroid hormone abnormality attributable to HDV. It may instead reflect subtle changes in the hypothalamic–pituitary–thyroid axis, hepatic hormone handling, systemic illness, or other unmeasured factors. Longitudinal studies incorporating thyroid antibodies and additional thyroid-related biomarkers would be required to clarify the mechanism.

### 4.4. AFP, HCC, and the Biochemical Phenotype of HDV Infection

AFP was higher in the HBV–HDV group overall and remained higher among patients without HCC. In the non-HCC subgroup, AFP correlated positively with AST and GGT, and the association with HBV–HDV status persisted after multivariable adjustment. Because AFP is not tumor-specific and may increase with hepatocellular injury, inflammation, and regeneration, the higher AFP observed in non-HCC patients may reflect differences in the underlying hepatic biochemical phenotype rather than occult malignancy. This analysis does not establish a causal relationship between HDV and AFP production, but it reinforces the need to interpret AFP in the context of HCC status and the underlying liver disease [14,15].

### 4.5. Clinical and Biological Implications

The present findings emphasize that the metabolic phenotype of HBV–HDV coinfection may not be adequately characterized by conventional between-group laboratory comparisons alone. Most circulating metabolic markers were similar between HBV monoinfection and HBV–HDV coinfection, whereas quantitative viral analysis identified an HDV RNA–triglyceride association within the coinfected group. This pattern supports further investigation of viral activity as a potential modifier of host metabolic pathways.

This question is particularly relevant in the current HDV treatment era, in which HDV RNA is a central virological endpoint and substantial reductions in HDV RNA can be achieved with antiviral therapy such as bulevirtide [21]. The present retrospective data cannot determine whether suppression of HDV replication changes triglyceride concentrations. However, prospective studies incorporating serial HDV RNA measurements together with lipidomic or targeted metabolomic profiling could test whether changes in viral activity are accompanied by reproducible changes in host lipid metabolism. Such studies could also help determine whether the triglyceride association observed here represents an isolated circulating biomarker or part of a broader HDV-associated metabolic signature.

### 4.6. Strengths and Limitations

The strengths of this study include the evaluation of a defined HBsAg-positive transplant population and the simultaneous assessment of conventional metabolic parameters, glucose–insulin homeostasis, thyroid function, quantitative viral markers, and AFP/HCC status. The principal HDV RNA–triglyceride association was supported by both non-parametric correlation and multivariable regression and remained consistent in BMI- and diabetes-adjusted sensitivity models.

Several limitations should be considered. First, the retrospective, single-center design limits causal inference and generalizability. Second, the cohort consisted of liver transplant candidates with advanced liver disease, so the findings may not apply to patients with early or non-cirrhotic HBV–HDV infection. Third, quantitative HDV RNA was available for only 42 of 104 patients with HBV–HDV coinfection, and complete-case sample sizes were smaller still for triglyceride regression models. Although most measured clinical and metabolic characteristics were similar between patients with and without an available HDV RNA result, HCC prevalence differed (2.4% vs. 17.7%), raising the possibility of selection bias. The persistence of the HDV RNA–triglyceride association after exploratory adjustment for HCC is reassuring but cannot eliminate this concern, particularly because only one HCC case was represented in the HDV RNA-available group. The RNA-positive-only subgroup was also too small for a reliable fully adjusted model. These limitations increase uncertainty around effect estimates and the possibility of model overfitting. Fourth, the long study period may have introduced analytical heterogeneity because laboratory methods and reporting conventions evolved over time; although thyroid measurements were harmonized where necessary, residual heterogeneity cannot be excluded. Fifth, several potential metabolic confounders—including dietary intake, detailed body composition, lipid-lowering therapy, physical activity, and hepatic steatosis—were not incorporated into the analyses. Sixth, lipid and thyroid measurements and virological measurements were not necessarily obtained on the same day: lipid and thyroid parameters were selected from the 1-month pre-transplant window, whereas HBV DNA and HDV RNA could have been obtained within the preceding 3 months. Although the most recent available pre-transplant values were used, temporal variability between measurements may have introduced residual measurement heterogeneity. Finally, the study evaluated conventional circulating biochemical markers rather than comprehensive metabolomic or lipidomic profiles. Triglycerides should therefore be regarded as a clinically accessible metabolic parameter, not as a metabolomic signature. Because several secondary analyses were exploratory, the findings are hypothesis-generating and require replication in an independent cohort.

## 5. Conclusions

In this HBsAg-positive liver transplant cohort, conventional circulating lipid and biochemical parameters were broadly similar between HBV monoinfection and HBV–HDV coinfection, while selected endocrine and metabolic associations emerged after multivariable or quantitative viral analyses. Lower TSH was consistently associated with HBV–HDV coinfection, whereas the adjusted diabetes association was attenuated after accounting for liver disease severity. Most notably, higher quantitative HDV RNA was independently associated with higher triglyceride concentrations within the HBV–HDV group, including after adjustment for HBV DNA, MELD score, BMI, and diabetes. This finding identifies a potential viral–metabolic interaction that warrants prospective validation. Serial HDV RNA assessment combined with targeted metabolomics or lipidomics may help determine whether suppression of HDV replication is accompanied by measurable changes in host lipid metabolism.

## Figures and Tables

**Figure 1:**
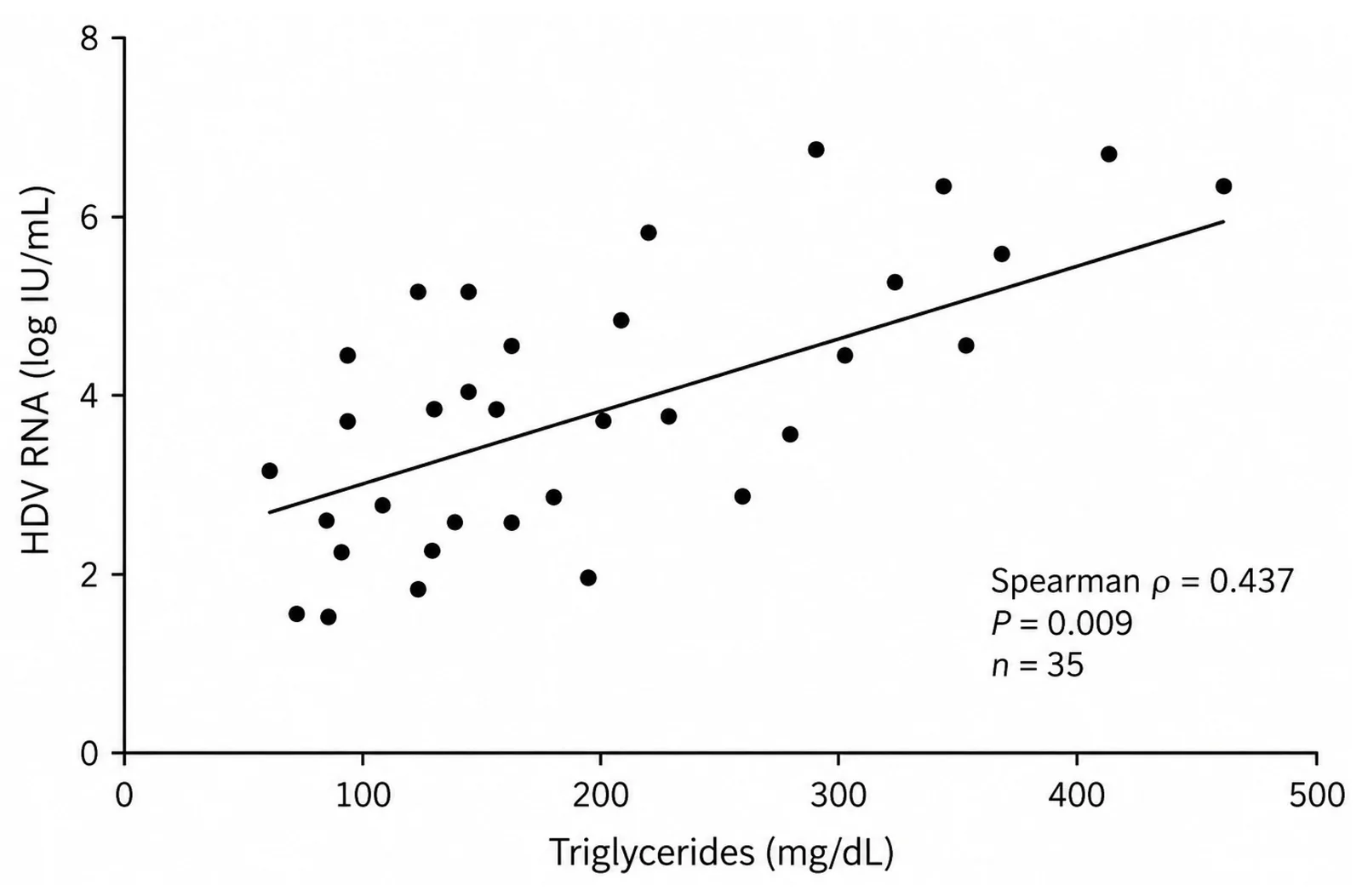
Association between HDV RNA level and triglyceride concentrations. Figure legend: Scatter plot showing the association between serum HDV RNA level and triglyceride concentration among patients with HBV–HDV coinfection. The correlation analysis included patients with available paired HDV RNA and triglyceride measurements (*n* = 35). HDV RNA values are displayed as log10(HDV RNA + 1) because of their markedly skewed distribution. Higher HDV RNA levels were positively correlated with triglyceride concentrations (Spearman ρ = 0.437, *p* = 0.009). In multivariable analysis adjusted for HBV DNA level, age, sex, and MELD score, higher HDV RNA remained independently associated with higher triglyceride concentrations (B = 5.42 per 1-log10 increase, 95% CI 1.84–9.00; *p* = 0.003). The fitted line is shown for visualization of the direction of the association.

**Figure 2:**
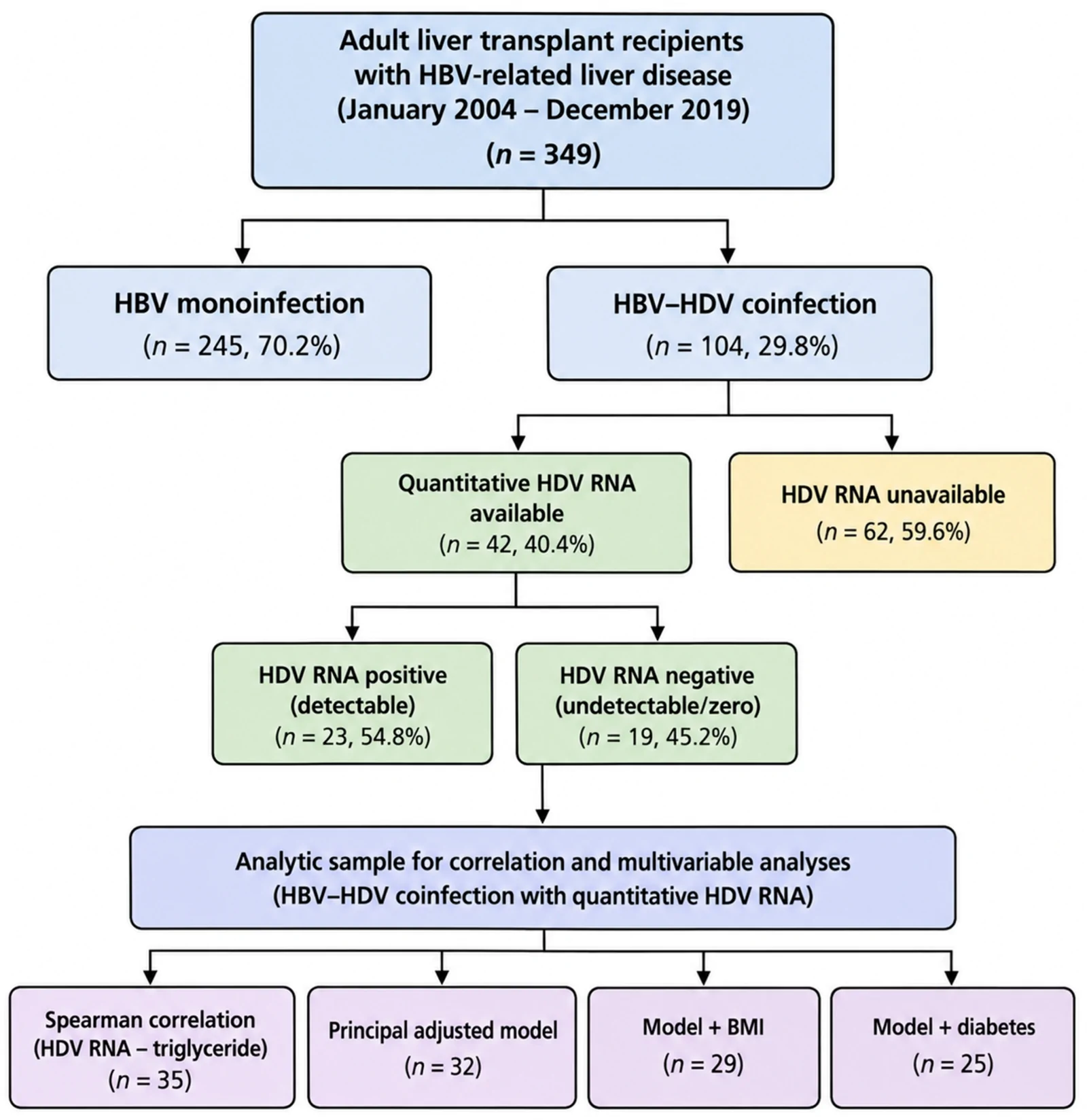
Patient flow and availability of quantitative HDV RNA for prespecified analyses.

**Table 1:** Clinical, metabolic, and endocrine characteristics of HBV monoinfection and HBV–HDV coinfection.

Variable	HBV	HBV-HDV	*p* Value
Age, years	53.00 (48.25–58.00) [*n* = 238]	48.00 (41.00–55.00) [*n* = 103]	<0.001
BMI, kg/m^2^	27.22 (24.69–29.56) [*n* = 213]	26.23 (24.37–29.41) [*n* = 102]	0.194
Fasting glucose, mg/dL	97.00 (87.75–114.25) [*n* = 220]	97.00 (86.50–117.00) [*n* = 103]	0.901
Diabetes mellitus	38/226 (16.8%)	23/94 (24.5%)	0.120
HbA1c, %	5.10 (4.60–5.70) [*n* = 193]	4.95 (4.40–5.90) [*n* = 84]	0.234
Insulin	14.91 (10.32–22.08) [*n* = 154]	18.95 (12.47–25.38) [*n* = 64]	0.070
HOMA-IR, recalculated	3.71 (2.44–6.61) [*n* = 150]	4.58 (2.86–6.95) [*n* = 64]	0.155
Total bilirubin, mg/dL	2.54 (1.40–4.49) [*n* = 228]	2.26 (1.12–4.01) [*n* = 102]	0.117
Creatinine, mg/dL	0.80 (0.70–1.00) [*n* = 228]	0.80 (0.60–0.90) [*n* = 104]	0.168
Total cholesterol, mg/dL	129.00 (95.00–156.00) [*n* = 205]	133.00 (100.50–164.50) [*n* = 87]	0.502
Triglycerides, mg/dL	70.00 (57.00–97.00) [*n* = 205]	76.00 (63.00–98.00) [*n* = 86]	0.170
TSH	1.71 (1.09–2.60) [*n* = 210]	1.44 (0.88–2.05) [*n* = 91]	0.011
FT3	3.56 (2.77–4.31) [*n* = 191]	3.80 (3.09–4.51) [*n* = 75]	0.088
FT4, standardized, pmol/L	15.32 (13.36–17.29) [*n* = 200]	15.57 (13.26–17.54) [*n* = 85]	0.672
MELD score	14.00 (11.00–18.00) [*n* = 205]	16.00 (13.00–18.00) [*n* = 99]	0.050

Continuous variables are presented as median (IQR); diabetes mellitus is presented as *n*/N (%). Between-group comparisons of continuous variables were performed using the Mann–Whitney U test. BMI and HOMA-IR were recalculated from the available source data, and FT4 values were standardized to pmol/L before analysis.

**Table 2:** Adjusted associations of HBV–HDV coinfection with metabolic and endocrine outcomes.

Outcome	*n*	Adjusted Effect	95% CI	*p* Value
Diabetes mellitus	274	aOR 2.075	1.057–4.076	0.034
HOMA-IR, recalculated	190	Ratio 1.243	0.989–1.562	0.062
Insulin	194	Ratio 1.205	0.988–1.470	0.065
TSH	263	Ratio 0.712	0.579–0.876	0.001
FT3	—	Ratio 1.077	0.953–1.218	0.236
FT4, standardized	—	Ratio 1.006	0.928–1.090	0.884

Models were adjusted for age, sex, MELD score, and recalculated BMI. Diabetes mellitus is reported as adjusted odds ratio; continuous outcomes were natural-log-transformed and reported as geometric mean ratios. Child–Pugh sensitivity analysis attenuated the diabetes association (aOR 1.890, 95% CI 0.919–3.885; *p* = 0.084). The source tables provide adjusted FT3 and FT4 effect estimates but do not state their model-specific *n* values; these are therefore not inferred here.

**Table 3:** Associations between HDV RNA level and metabolic parameters in HBV–HDV coinfection.

A. Correlations between HDV RNA and selected metabolic parameters				
Metabolic parameter	*n*	Spearman rho		*p* value
Total bilirubin	40	0.047		0.772
Creatinine	42	−0.065		0.684
Fasting glucose	42	0.328		0.034
Total cholesterol	35	0.115		0.511
Triglycerides	35	0.437		0.009
B. Multivariable association between HDV RNA and triglyceride concentration				
Model	*n*	B per 1-log10 increase in HDV RNA	95% CI	*p* value
Main model: HBV DNA + age + sex + MELD	32	5.42	1.84–9.00	0.003
Sensitivity: main model + BMI	29	5.33	1.53–9.14	0.006
Sensitivity: main model + diabetes mellitus	25	5.32	0.23–10.41	0.041

Part A used Spearman rank correlation. In Part B, HDV RNA and HBV DNA were entered as log10(value + 1). B represents the change in triglyceride concentration for each 1-log10 increase in HDV RNA. HC3-robust standard errors were used. These analyses are exploratory and do not establish causality.

**Table 4:** Detailed AFP analyses according to HCC status in HBV monoinfection and HBV–HDV coinfection.

A. AFP according to HCC status				
Population	HBV	HBV-HDV		*p* value
All patients	6.02 (3.27–19.98) [*n* = 218]	9.80 (3.82–49.81) [*n* = 99]		0.029
HCC absent	5.29 (2.93–11.19) [*n* = 128]	12.59 (4.00–55.47) [*n* = 87]		<0.001
HCC present	11.25 (4.14–33.09) [*n* = 90]	5.31 (2.95–8.39) [*n* = 12]		0.060
B. Correlations of AFP with liver-related variables among patients without HCC				
Variable	*n*	Spearman rho		*p* value
GGT	212	0.245		<0.001
AST	213	0.226		<0.001
ALT	202	0.129		0.067
ALP	210	0.104		0.134
Albumin	214	−0.075		0.277
INR	207	−0.070		0.316
Total bilirubin	212	−0.044		0.528
Platelets	194	0.041		0.573
MELD score	189	0.021		0.774
C. Multivariable analyses of AFP among patients without HCC				
Adjustment model	*n*	HBV-HDV/HBV AFP ratio	95% CI	*p* value
Age + sex + MELD + BMI	188	2.788	1.822–4.267	<0.001
Age + sex + BMI + AST + platelets	190	2.861	1.906–4.297	<0.001
Age + sex + BMI + AST + total bilirubin + INR + albumin + platelets	183	3.030	1.948–4.714	<0.001
Adjustment model	*n*	HBV-HDV/HBV ratio	95% CI	*p* value
Age + sex + BMI + platelets	273	0.702	0.568–0.866	<0.001
Age + sex + BMI + albumin	275	0.712	0.575–0.883	0.002
Age + sex + BMI + sodium	274	0.691	0.557–0.856	<0.001
Age + sex + BMI + platelets + albumin + sodium	272	0.697	0.562–0.863	<0.001
Age + sex + BMI + MELD + platelets + albumin	263	0.726	0.590–0.893	0.002

AFP values are median (IQR). Part B uses Spearman correlations. Part C uses log-transformed AFP with HC3-robust standard errors; these AFP analyses are exploratory.

## Data Availability

The data presented in this study are available from the corresponding author upon reasonable request.

## Supplementary Materials

The following supporting information can be downloaded at https://www.mdpi.com/article/10.3390/metabo16100709/s1, Figure S1: Sensitivity Analyses of the HDV RNA–Triglyceride Association; Table S1: Sensitivity Analyses of the Association Between HBV–HDV Coinfection and TSH; Table S2: Comparison of HBV–HDV Patients With and Without Available Quantitative HDV RNA. Supplementary files are provided separately.
