# Supplementary material for: Metabolic and Thyroid Alterations in HBV Monoinfection and HBV–HDV Coinfection: Insights from a Liver Transplant Cohort
Source: Metabolites. 2026 Sep 24;16(10):709. doi: 10.3390/metabo16100709 (PMC13706769; doi:10.3390/metabo16100709)
Supplement: Supplementary file 1 [file metabolites-16-00709-s001.zip › metabolites-4536937-supplementary.pdf]

Supplementary Figure S1. Sensitivity Analyses of the HDV RNA–Triglyceride Association.

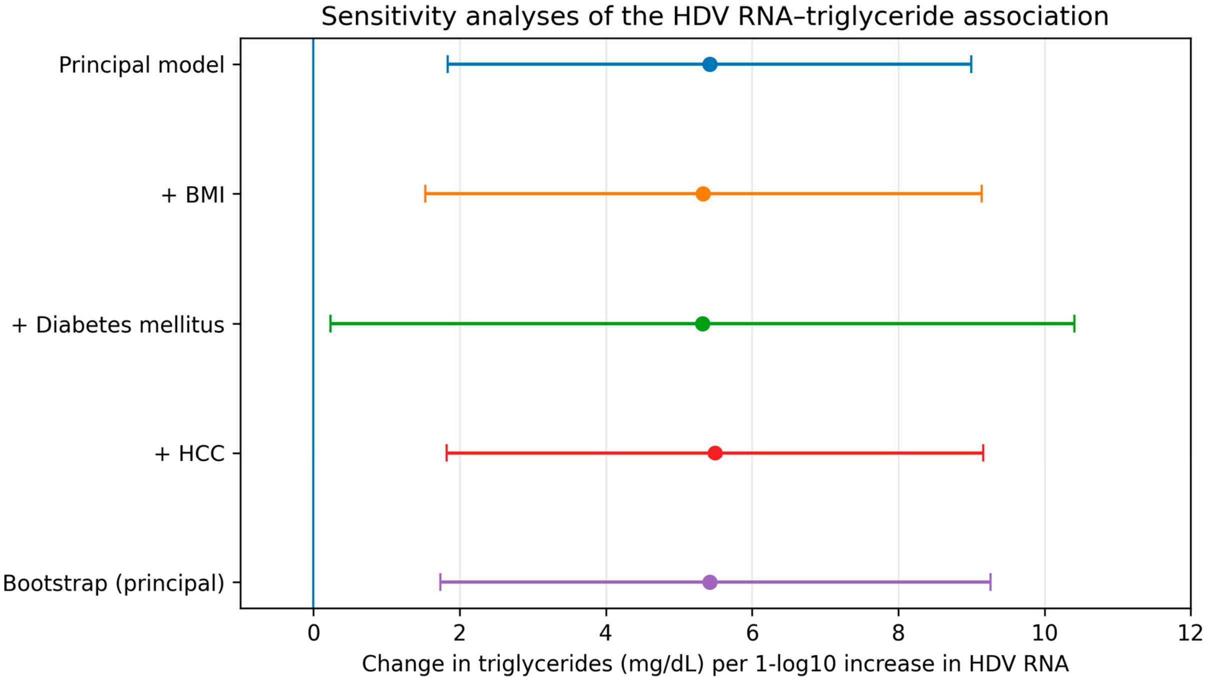

Legend. Forest plot showing regression coefficients for the association between quantitative HDV RNA and triglyceride concentration. Points represent the estimated change in triglycerides (mg/dL) per 1-log10 increase in HDV RNA, and horizontal lines represent 95% confidence intervals. The principal model was adjusted for HBV DNA, age, sex, and MELD score. Sensitivity models additionally included BMI, diabetes mellitus, or HCC. The bootstrap result represents the 5000-resample percentile 95% confidence interval for the principal-model coefficient. The direction of association remained positive across all displayed sensitivity analyses. The log-transformed triglyceride sensitivity analysis also remained significant (P = 0.0051), corresponding to an estimated 6.5% increase in triglycerides per 1-log10 increase in HDV RNA (95% CI approximately 1.9%–11.3%).

Supplementary Table S1. Sensitivity Analyses of the Association Between HBV–HDV Coinfection and TSH.

Adjustment model	n	HBV-HDV/HBV ratio	95% CI	P value
Age + sex + BMI + platelets	273	0.702	0.568-0.866	<0.001
Age + sex + BMI + albumin	275	0.712	0.575-0.883	0.002
Age + sex + BMI + sodium	274	0.691	0.557-0.856	<0.001
Age + sex + BMI + platelets + albumin + sodium	272	0.697	0.562-0.863	<0.001
Age + sex + BMI + MELD + platelets + albumin	263	0.726	0.590-0.893	0.002

Note. TSH was natural-log transformed; ratios compare HBV–HDV coinfection with HBV monoinfection. HC3 robust standard errors were used.

Supplementary Table S2. Comparison of HBV–HDV Patients With and Without Available Quantitative HDV RNA.

Variable	RNA available n	RNA available	RNA missing n	RNA missing	P value

Age, years	42	46.00 (40.50–55.00)	61	49.00 (41.00–54.00)	0.506
MELD score	42	15.00 (13.00–18.00)	57	16.00 (13.00–18.00)	0.744
Triglycerides, mg/dL	35	73.00 (62.50–92.50)	51	79.00 (64.00–102.00)	0.379
BMI, kg/m²	38	26.60 (24.92–29.48)	34	26.00 (23.50–30.32)	0.672
TSH	36	1.33 (0.85–2.07)	55	1.50 (0.89–2.04)	0.792
FT3	26	3.75 (2.99–4.25)	49	3.80 (3.20–4.52)	0.632
HbA1c, %	28	5.10 (4.28–5.83)	56	4.90 (4.47–6.05)	0.494
Diabetes mellitus	35	9/35 (25.7%)	59	14/59 (23.7%)	1.000
HCC	42	1/42 (2.4%)	62	11/62 (17.7%)	0.025

Note. Continuous variables are presented as median (IQR) and were compared using the Mann–Whitney U test. Categorical variables are presented as n/N (%) and were compared using Fisher's exact test. Sample sizes vary because missing values were not imputed and each comparison used available cases. HCC was less frequent among patients with an available quantitative HDV RNA result; other listed comparisons were not statistically significant. Abbreviations: BMI, body mass index; FT3, free triiodothyronine; HCC, hepatocellular carcinoma; MELD, Model for End-Stage Liver Disease; TSH, thyroid-stimulating hormone.
